# Can proximal gastrectomy with double-tract reconstruction replace total gastrectomy? a meta-analysis of randomized controlled trials and propensity score-matched studies

**DOI:** 10.1186/s12876-024-03323-7

**Published:** 2024-07-23

**Authors:** Guangxu Zhu, Xuguang Jiao, Shengjie Zhou, Qingshun Zhu, Lei Yu, Qihang Sun, Bowen Li, Hao Fu, Jie Huang, Wei Lang, Xiaomin Lang, Shengyong Zhai, Jinqiu Xiong, Yanan Fu, Chunxiao Liu, Jianjun Qu

**Affiliations:** 1 Department of General Surgery, Weifang People’s Hospital, Weifang, Shandong China; 2 Department of Anesthesiology, Weifang People’s Hospital, Weifang, Shandong Weifang China; 3 Weifang Medical University, Weifang, Shandong China; 4 Medical Administration Dept, Weifang People’s Hospital, Weifang, Shandong China; 5 Department of General Surgery, the First Affiliated Hospital of Nanjing Medical University, Nanjing, Jiangsu China

**Keywords:** Gastric cancer, Proximal gastrectomy with double-tract reconstruction, Total gastrectomy with Roux-en-Y reconstruction, Quality of life, Nutritional status, Oncologic outcomes, Meta-analysis

## Abstract

**Background:**

According to the 5th edition of the Japanese Guidelines for the Treatment of Gastric Cancer, proximal gastrectomy is recommended for patients with early upper gastric cancer who can retain the distal half of the residual stomach after R0 resection. However, a large number of recent clinical studies suggest that surgical indications for proximal gastrectomy in the guidelines may be too narrow. Therefore, this meta-analysis included patients with early and advanced gastric cancer and compared short- and long-term postoperative outcomes between the two groups. At the same time, we only had high-quality clinical studies such as propensity score-matched studies and randomized controlled trials, which made our research more authentic and credible.

**Methods:**

Data were retrieved from PubMed, EMBASE, Medline, and Cochrane Library up to June 2023, and included treatment outcomes after proximal gastrectomy with double-tract reconstruction and total gastrectomy with Roux-en-Y reconstruction. The primary results were Early-phase complications(Anastomotic leakage, Anastomotic bleeding, Abdominal abscess, Abdominal infection, Pulmonary infection, Incision infection, Intestinal obstruction, Dumping syndrome, Pancreatic fistula), Late-phase complications(Intestinal obstruction, Anastomosis stricture, Dumping syndrome, Reoperation, Internal hernia, Incidence of endoscopic gastroesophageal reflux), Serious complications (≥ Grade III C-D score), Quality of life[Gastroesophageal reflux symptom evaluation (Visick score)(≥ III), Los Angeles classification(C or D)], Nutritional status(Hemoglobin, Receipt of vitamin B12 supplementation), Oncologic Outcomes(The 5-year overall survival rates). Secondary outcomes were surgical outcomes (Operative time, Estimated blood loss, Postoperative hospital stay, Number of harvested lymph nodes, Gas-passing, Postoperative mortality).The Cochrane risk-of-bias tool and Newcastle‒Ottawa scale were used to assess the quality of the included studies.

**Results:**

After screening, 11 studies were finally included, including 1154 patients. Results from the combined literature showed that total gastrectomy had a significant advantage over proximal gastrectomy with double-tract reconstruction in mean operating time (MD = 4.92, 95% CI: 0.22∼9.61 *P* = 0.04). However, meta-analysis results showed that Hemoglobin (MD = 7.12, 95% CI:2.40∼11.84, *P* = 0.003) and Receipt of vitamin B12 supplementation (OR = 0.12, 95% CI:0.05∼0.26, *P* < 0.00001) in the proximal gastrectomy with double-tract reconstruction group were better than those in the total gastrectomy with Roux-en-Y reconstruction group. There is no significant difference between the proximal gastrectomy with double-tract reconstruction and the total gastrectomy with Roux-en-Y reconstruction group in Early-phase complications(OR = 1.14,95% CI:0.79∼1.64, *P* = 0.50), Late-phase complications(OR = 1.37,95% CI:0.78∼2.39, *P* = 0.27), Gastroesophageal reflux symptom evaluation (Visick score)(≥ III)(OR = 0.94,95% CI:0.14∼1.07 *P* = 0.07), Los Angeles classification(C or D)(OR = 0.33,95% CI:0.01∼8.21, *P* = 0.50), the 5-year overall survival rates (HR = 1.01, 95% CI: 0.83 ~ 1.23, *P* = 0.89).

**Conclusion:**

Proximal gastrectomy with double-tract anastomosis is a safe and feasible treatment for upper gastric carcinoma. However, the operating time was slightly longer in the proximal gastrectomy with double-tract group compared to the total gastrectomy with Roux-en-Y group. The two groups were comparable to the total gastrectomy with Roux-en-Y group in terms of serious complications (≥ Grade III C-D score), early-phase complications, late-phase complications, and quality of life. Although the scope of proximal gastrectomy is smaller than that of total gastrectomy, it does not affect the 5-year survival rate, indicating good tumor outcomes for patients. Compared to total gastrectomy with Roux-en-Y group, proximal gastrectomy with double-tract reconstruction had higher hemoglobin levels, lower probability of vitamin B12 supplementation, and better long-term efficacy. In conclusion, proximal gastrectomy with double-tract reconstruction is considered one of the more rational surgical approaches for upper gastric cancer.

## Introduction

In recent years, there has been an increasing trend in the incidence of upper gastric cancer, including esophagogastric junction cancer, gastric fundus cancer, and parts of upper gastric body cancer [1]. The surgical treatment of tumors in this part has been a topic of controversy. Proximal gastrectomy presents a major challenge due to the high rate of severe postoperative complications, particularly reflux esophagitis. This condition can lead to severe reflux, chest pain, and anorexia in patients, significantly reducing their quality of life after surgery. In 1988, AIKOU et al. first reported the application of double-tract reconstruction after proximal gastrectomy and achieved good short-term results for the patient after surgery [2]. Compared with total gastrectomy combined with Roux-en-Y reconstruction (TG), proximal gastrectomy with double-tract reconstruction (PG-DTR) is more aligned with the physiological and anatomical requirements of the digestive tract. Preserving distal gastric stumps can increase food storage for patients and effectively reduce long-term complications such as iron deficiency anemia and malnutrition. Double-tract anastomosis with preservation of the gastric antrum is beneficial for preventing reflux esophagitis and dumping syndrome while also reducing the incidence of Roux syndrome because the residual stomach plays a role in both storage capacity and feeding through a double-tract path.

Recently, [3] conducted a meta-analysis comparing proximal gastrectomy with double-tract reconstruction and total gastrectomy combined with Roux-en-Y reconstruction in terms of short-term and long-term outcomes. However, the study only included 1 randomized controlled trial and 13 observational studies, which may limit the credibility of the findings. Furthermore, subgroup analysis by reference type (RCT vs. non-RCT) was not performed, adding to the limitations of the study. Given the recent publication of numerous high-quality randomized controlled trials and propensity score-matched studies on this topic, there is a need for an updated and comprehensive systematic review and meta-analysis. In our systematic review and meta-analysis, we specifically included high-quality papers such as RCTs and PSMs to ensure the credibility of our findings. Additionally, our meta-analysis incorporated more comprehensive clinical data including complication severity (≥ Grade III C-D score), early-phase complications, late-phase complications, and quality of life measures (Gastroesophageal reflux symptom evaluation [Visick score] (≥ III), Los Angeles classification [C or D]) to provide a more complete analysis.

## Methods

This meta-analysis of included studies (propensity-score-matched studies, randomized controlled trials )was based on reporting items for systematic study reviews and meta-analysis statements (PRISMA) [4].

### Literature retrieval

Literature was retrieved from EMBASE, PubMed, Medline, and Cochrane Library. Search keywords were (“stomach” OR “gastric”) AND (“neoplasm” OR “cancer” OR “tumor” OR “carcinoma” ) AND (“dual-channel” OR “double tract reconstruction” OR“double tract anastomosis”). The search ended on 2023.06.

### Inclusion and exclusion criteria

Inclusion criteria were determined according to the PICOS approach as follows: P: Patients who were pathologically diagnosed with gastric cancer and underwent gastrectomy; I: Proximal gastrectomy with double-tract reconstruction; C: Total gastrectomy; O: Short- and long-term outcomes; S: Comparative studies including PSMs and RCTs. Exclusion criteria were as follows: (1) Literature that does not contain data from both proximal gastrectomy with double-tract reconstruction and total gastrectomy comparative studies; (2) Clinical study of preoperative neoadjuvant chemotherapy; (3) Repeated published clinical studies; (4) Clinical studies in which the full text cannot be found, there is insufficient raw data, or the required data is incomplete and cannot be analyzed.

### Data extraction and quality assessment

Screening is conducted by two professionals strictly according to inclusion and exclusion criteria, with independent cross-checks completed. In cases of controversial literature, a third party is consulted to decide whether to include the disputed research. Data extraction was carried out for eligible literature, including author, study period, country, study design, sample size, age, sex, TNM stage, and follow-up duration. The primary results included early-phase complications (such as anastomotic leakage, anastomotic bleeding, abdominal abscesses, and pulmonary infection), late-phase complications (including intestinal obstruction and reoperation), serious complications (≥ Grade III C-D score), quality of life assessments (e.g., Gastroesophageal reflux symptom evaluation - Visick score ≥ III), nutritional status indicators (hemoglobin levels and receipt of vitamin B12 supplementation), and oncologic outcomes such as 5-year overall survival rates. Secondary outcomes focused on surgical factors such as operative time and postoperative mortality. The Newcastle‒Ottawa scale was used to assess the quality of PSM studies while the Cochrane risk-of-bias tool was utilized for assessing RCT studies [5].

### Statistical analysis

We utilized ReviewManager 5.4 software and Stata 15.1 software for our meta-analysis. Weighted mean difference (WMD) was employed to assess the operative time, estimated blood loss, postoperative hospital stay, and operative time of the two groups in this meta-analysis. Additionally, we used WMD to analyze the number of lymph nodes harvested and the combined effect size of Gas-passing and Hemoglobin. We used odds ratio (OR) to evaluate Gastroesophageal reflux symptoms (Visick score)(≥ III), Los Angeles classification (C or D), Receipt of vitamin B12 supplementation, Oncologic Outcomes(the 5-year overall survival rates), Early-phase complications(Anastomotic leakage, Anastomotic bleeding, Abdominal abscess, Abdominal infection, Pulmonary infection, Incision infection, Intestinal obstruction, Dumping syndrome, Pancreatic fistula), and Late-phase complications(Intestinal obstruction, Anastomosis stricture, Dumping syndrome, Reoperation, Internal hernia, The incidence of endoscopic gastroesophageal reflux). The heterogeneity among study results was assessed using the I^2^ value. I^2^ value exceeding 50% indicates significant heterogeneity and warrants a random effects model; conversely, I^2^ value below 50% suggests moderate to low heterogeneity and calls for a fixed effect model.If significant heterogeneity between studies was found, sensitivity analysis was conducted to address it.The funnel plot was utilized to test publication bias.A P-value < 0.05 indicates statistical significance.

## Results

### Inclusion of literature and quality assessment

**Fig. 1:**
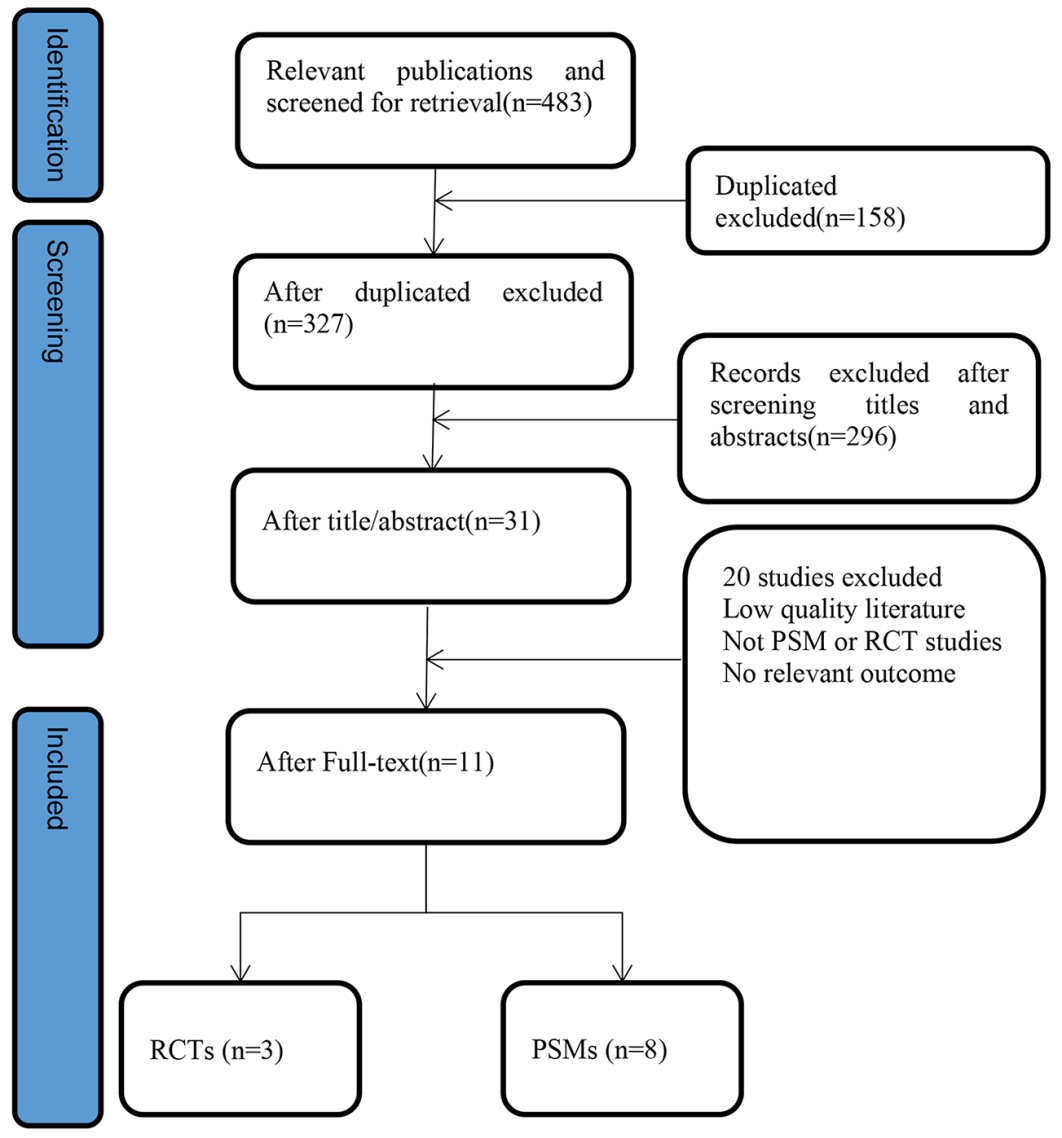
Flow chart of literature search process

**Fig. 2:**
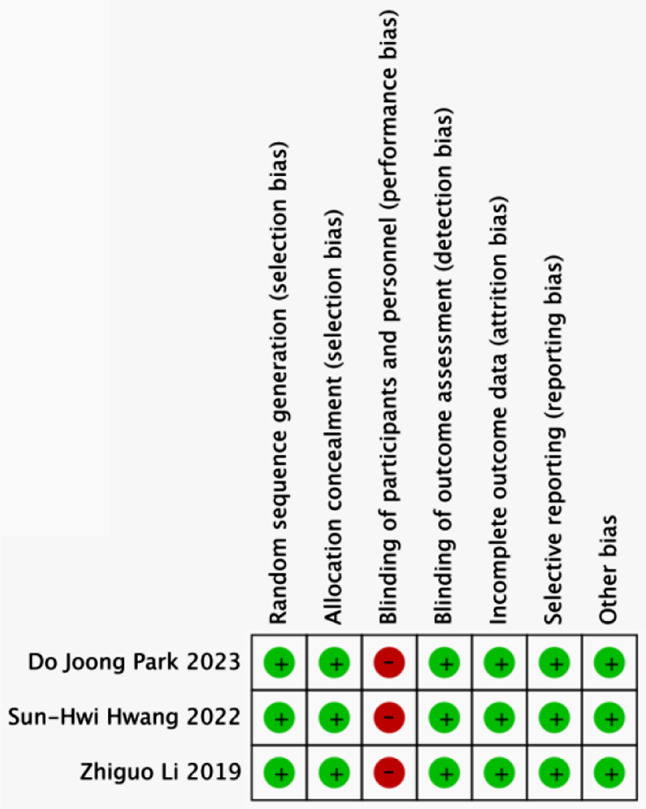
Methodological quality of randomized controlled studies

We initially retrieved 1154 papers from English databases and ultimately included 11 papers through literature screening based on inclusion and exclusion criteria (Fig. 1). Among the included literature, 3 papers were RCT studies, and 8 were PSM studies. All articles were published between 2019 and 2023, with sample sizes ranging from 16 to 202. Of these studies, two were conducted in Japan, four in Korea, and five in China. The results of the literature quality assessment are presented in Table 1; Fig. 2.

**Table 1:** Characteristics of the included studies

Study	Year	Study period	Country	Study design	Group	Sample size	Age(Y)	Sex(M/F)	BMI(kg/m2)	TNM stage(I/II/III)	Follow-up duration(months)	
RCTs(*n* = 3)												
Do Joong Park [6]	2023	2016–2018	Korea	RCT	PG-DTR	68	56.7 ± 10.4	39/29	24.5 ± 2.8	65/3/0	24.6 (2.9–35.3)	
					TG	69	61.3 ± 11.3	48/21	24.3 ± 3.0	62/7/0	24.6 (2.9–35.3)	
Sun-Hwi Hwang [7]	2022	2016–2018	Korea	RCT	PG-DTR	68	56.7 ± 10.4	39/29	NA	65/2/1	NA	
					TG	69	61.4 ± 11.3	48/21	NA	62/6/1	NA	
Zhiguo Li [8]	2019	2015–2017	China	RCT	PG-DTR	103	< 60 23(22.33) ≥ 60 80(77.67)	90/13	NA	87/16/0	30	
					TG	99	< 60 18(18.18) ≥ 60 18 (18.18)	82/17	NA	78/21/0	30	
PSMs(*n* = 8)												NOS
DONG JIN KIM [9]	2016	2009–2014	Korea	PSM	PG-DTR	17	64.7 ± 9.9	14/3	24.2 ± 3.8	16/1/0	18	7
					TG	17	60.9 ± 12.9	10/7	23.4 ± 5	15/2/0	18	
Fei Ma [10]	2020	2010–2018	China	PSM	PG-DTR	86	60.5 ± 8.2	79/7	NA	31/27/28	48 [26–72]	8
					TG	86	62.7 ± 10.5	76/10	NA	35/22/29	48 [26–72]	
Hyo Jung Ko [11]	2019	2008–2016	Korea	PSM	PG-DTR	52	61.5 ± 12.3	35/17	23.7 ± 3.1	45/5/2	22.7 ± 15.4	8
					TG	52	63.0 ± 9.2	35/17	23.4 ± 2.9	40/7/5	36.3 ± 23.1	
Kotaro Kimura [12]	2021	2011–2018	Japan	PSM	PG-DTR	8	70.4 (55–77)	8/0	22.9 (19.3–24.9)	8/0/0	24	8
					TG	8	68.3 (45–83)	8/0	23.7 (20.8–29.8)	8/0/0	24	
Linjun Wang [13]	2020	2016–2017	China	PSM	PG-DTR	12	55.58 ± 4.100	6/6	23.29 ± 0.7648	10/2/0	12	7
					TG	24	58.38 ± 1.825	15/9	23.5 ± 0.6898	22/2/0	12	
Reo Sato [14]	2021	2013–2019	Japan	PSM	PG-DTR	75	68.8 ± 10.2	59/16	22.4 [15.8–32.2]	60/11/4	36.3[22.2–51.0]	8
					TG	75	66.2 ± 11.8	51/24	22.8 [16.0–34.8]	44/16/12	36.3[22.2–51.0]	
Xiaoming Ma [15]	2022	2013–2018	China	PSM	PG-DTR	33	≤ 60 10; >60 23	5/28	NA	14/9/10	54 (2–94)	9
					TG	33	≤ 60 12; >60 21	5/28	NA	10/12/11	61 (6–95)	
Zhi Guo Li [16]	2023	2012–2015	China	PSM	PG-DTR	50	65.8 ± 4.5	40/10	24.1 ± 1.8	50/0/0	60	8
					TG	50	66.9 ± 3.6	40/10	23.8 ± 2.0	50/0/0	60	

#### Results of the meta-analysis of the secondary outcomes

**Fig. 3:**
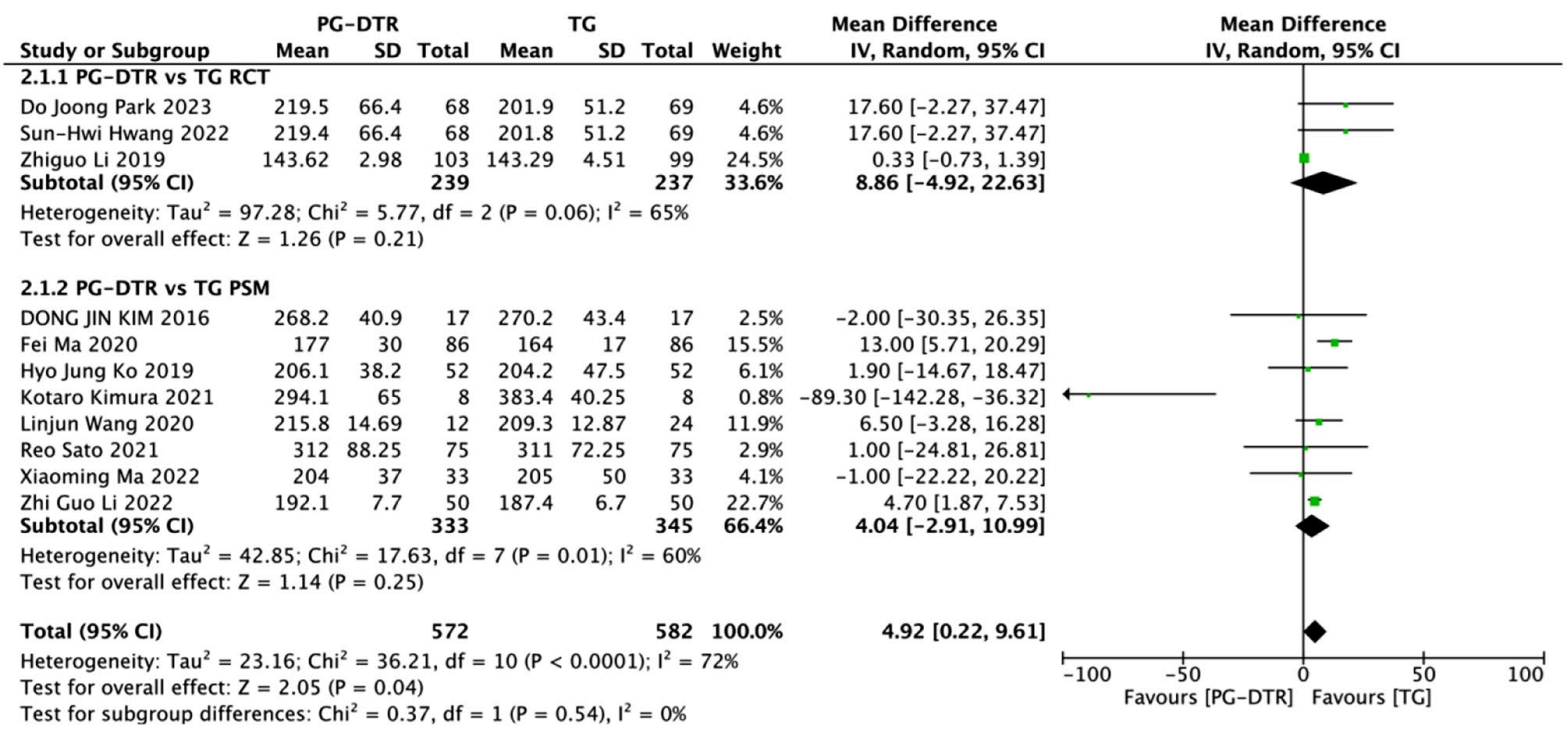
Forest plots of comparision between PG-DTG and TG-RY on mean operation time

A total of 1154 patients in 11 papers were meta-analyzed in terms of mean operating time [6–16]. The results from the combined literature showed that TG had a significant advantage over PG-DTR in mean operating time. (MD = 4.92, 95% CI: 0.22∼9.61 *P* = 0.04). Heterogeneity test (*P* < 0.00001, I^2^ = 72%). (Fig. 3).

**Fig. 4:**
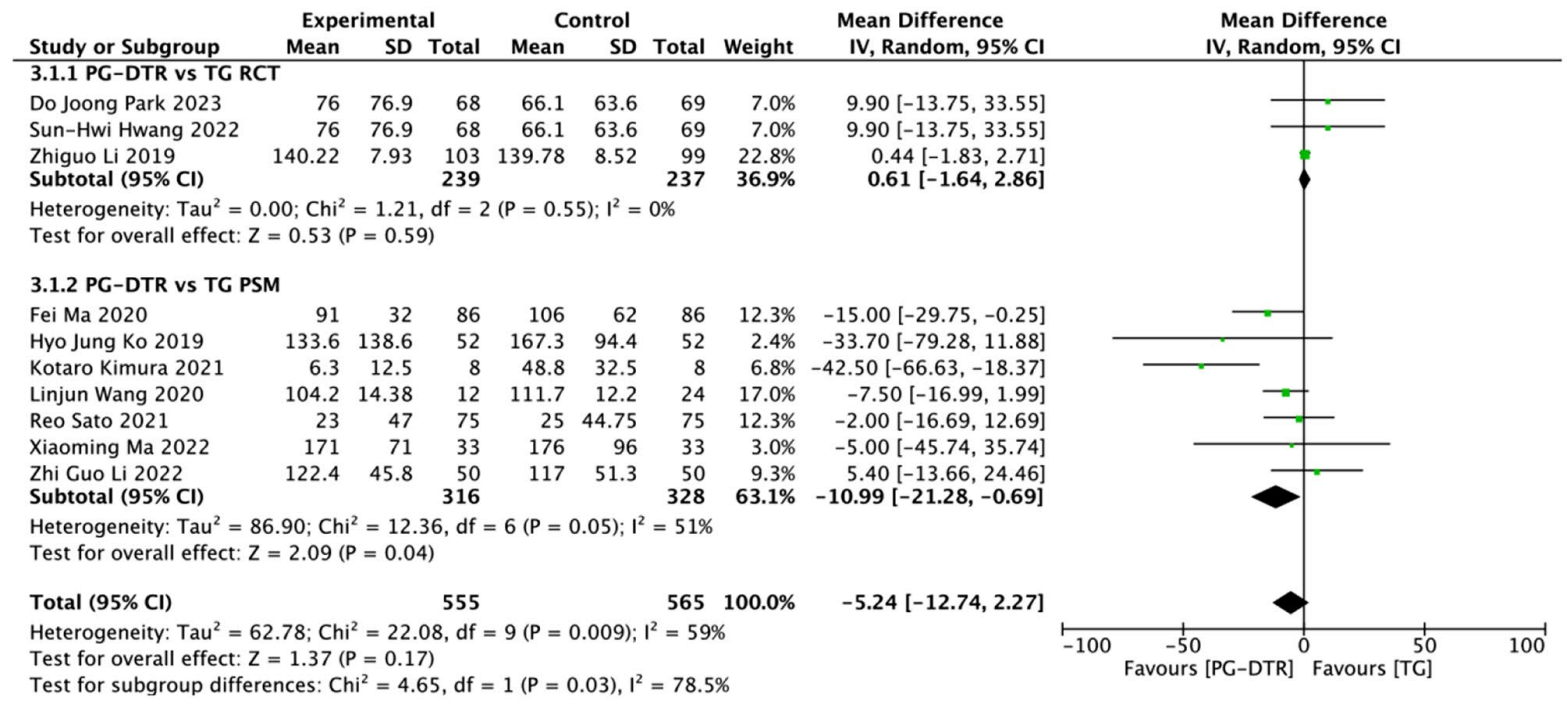
Forest plots of comparision between PG-DTG and TG-RY on estimated blood loss

A total of 1120 patients in 10 papers were meta-analyzed in terms of estimated blood loss [6–8, 10–16]. The results from the combined literature showed that PG-DTR did not have a significant advantage over TG in estimated blood loss (MD=-5.24, 95% CI: -12.74∼2.27, *P* = 0.17). Heterogeneity test (*P* < 0.009, I^2^ = 59%). (Fig. 4).

**Fig. 5:**
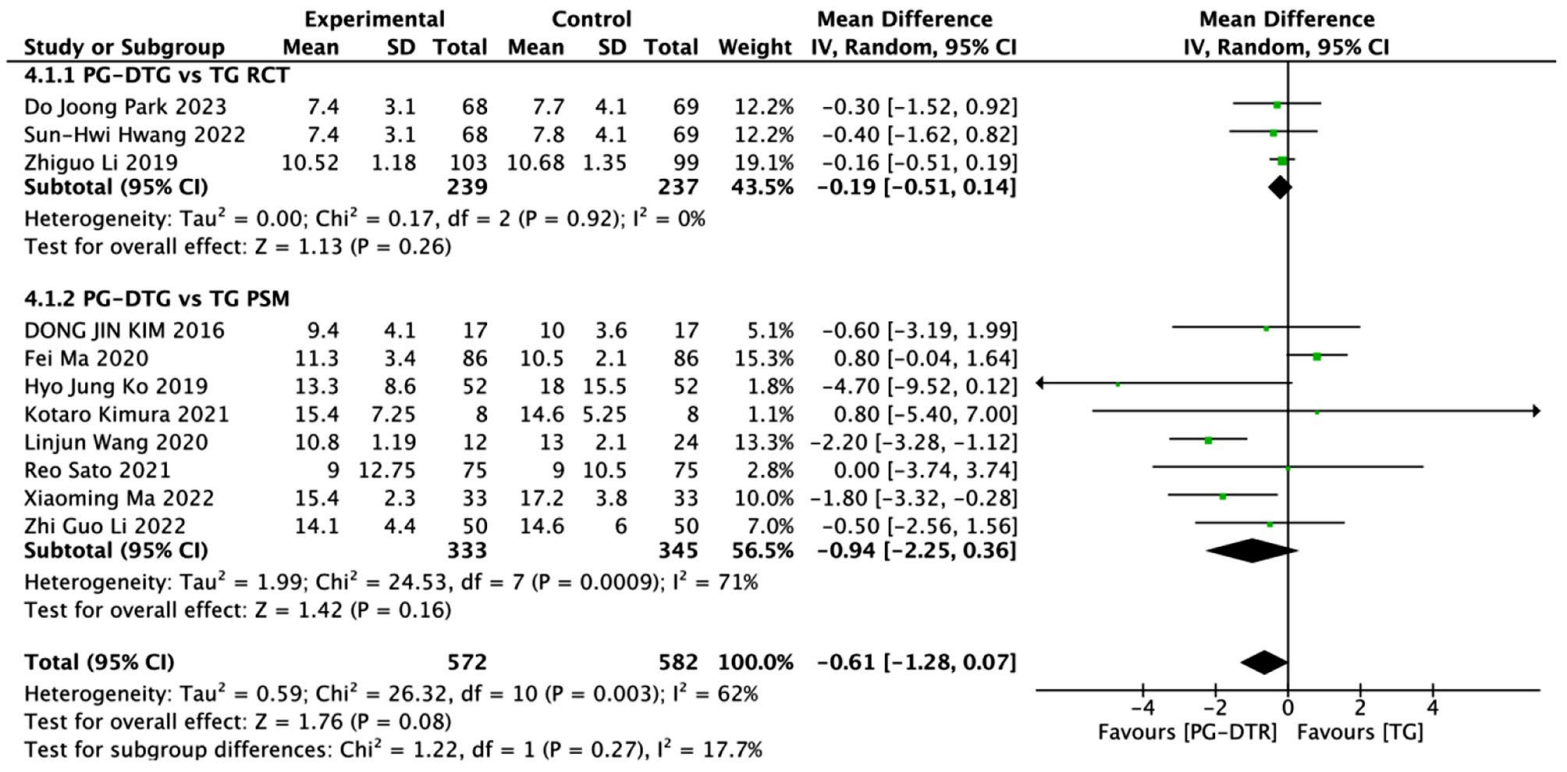
Forest plots of comparision between PG-DTG and TG-RY on postoperative hospital stay

A total of 1154 patients in 11 papers were meta-analyzed in terms of hospital stay after surgery [6–16]. The results from the combined literature showed that PG-DTR did not have a significant advantage over TG in hospital stay after surgery (MD=-0.61, 95% CI:-1.28 ~ 0.07, *P* = 0.08). Heterogeneity test (*P* = 0.003, I^2^ = 62%). (Fig. 5).

**Fig. 6:**
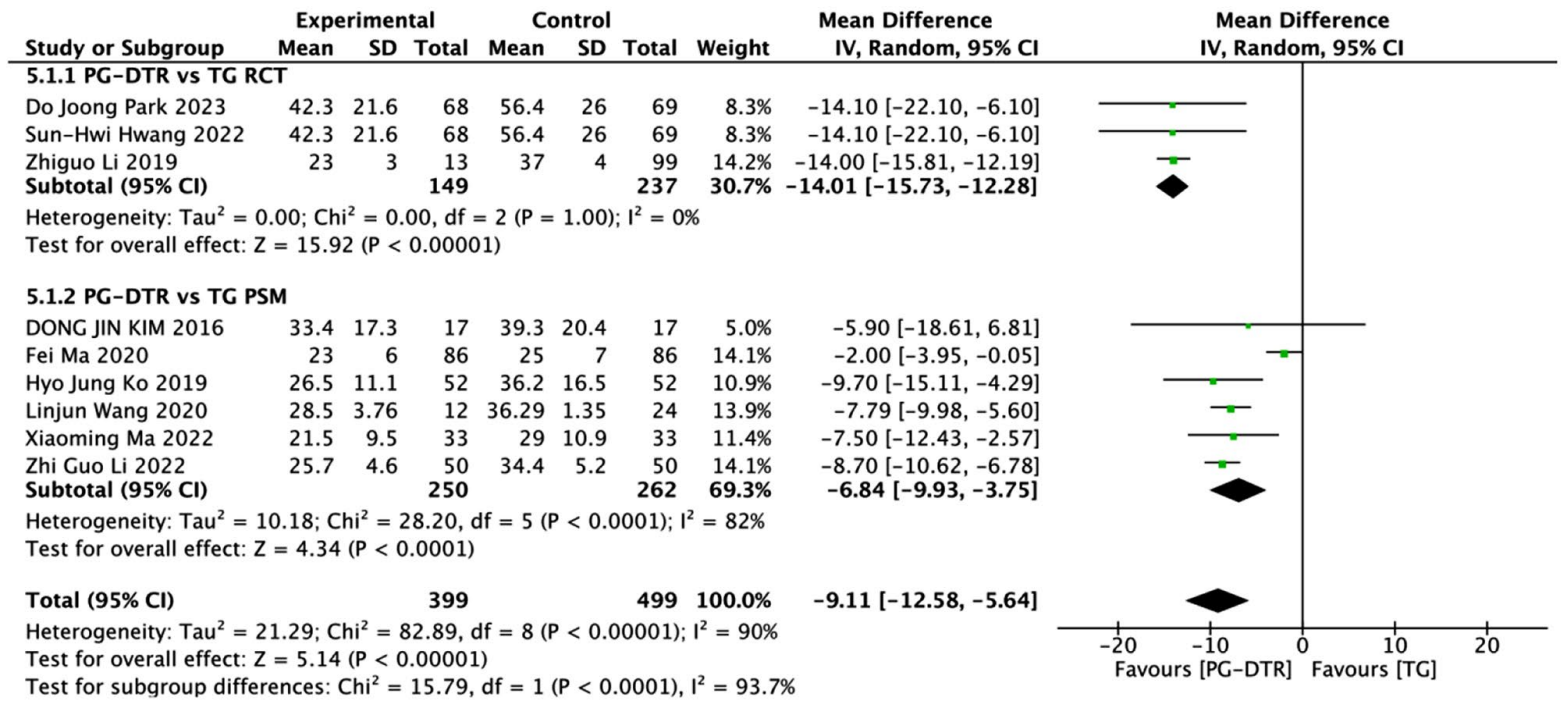
Forest plots of comparision between PG-DTG and TG-RY on number of harvested lymph nodes

A total of 898 patients in 9 papers were meta-analyzed in terms of the retrieved number of lymph nodes [6–11, 13, 15, 16]. In this meta-analysis, it was determined that the PG-DTR group obtained fewer lymph nodes compared to the TG group (MD=-9.11, 95% CI:-12.58~-5.64,*P* < 0.00001) .Heterogeneity test (*P* < 0.00001, I2 = 90%). (Fig. 6)

**Fig. 7:**
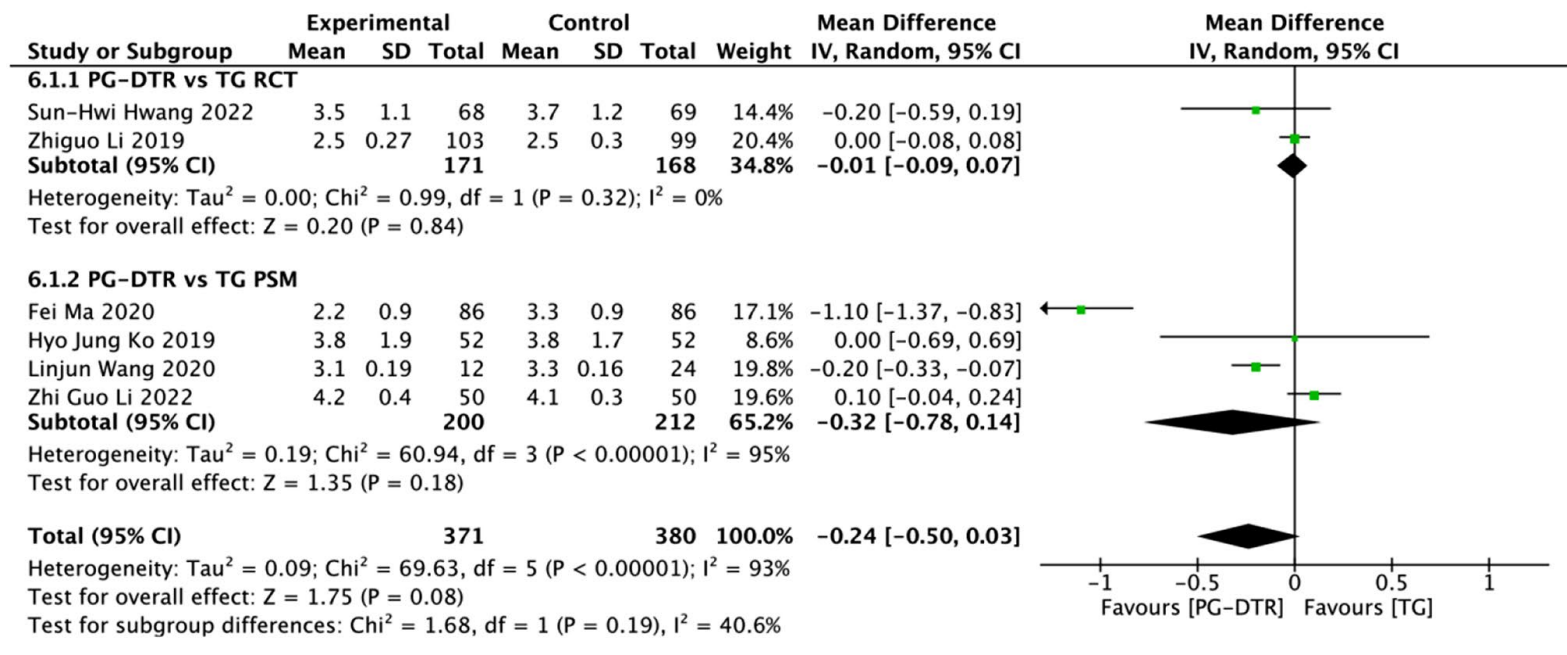
Forest plots of comparision between PG-DTG and TG-RY on gas-passing, days

A total of 751 patients in 6 papers were meta-analyzed in terms of time to Gas-passing [7, 8, 10, 11, 13, 16]. The results from the combined literature showed that PG-DTR did not have a significant advantage over TG in time to Gas-passing (MD=-0.24, 95% CI:-0.50 ~ 0.03, *P* = 0.08). Heterogeneity test (*P* < 0.00001, I^2^ = 93%). (Fig. 7).

**Fig. 8:**
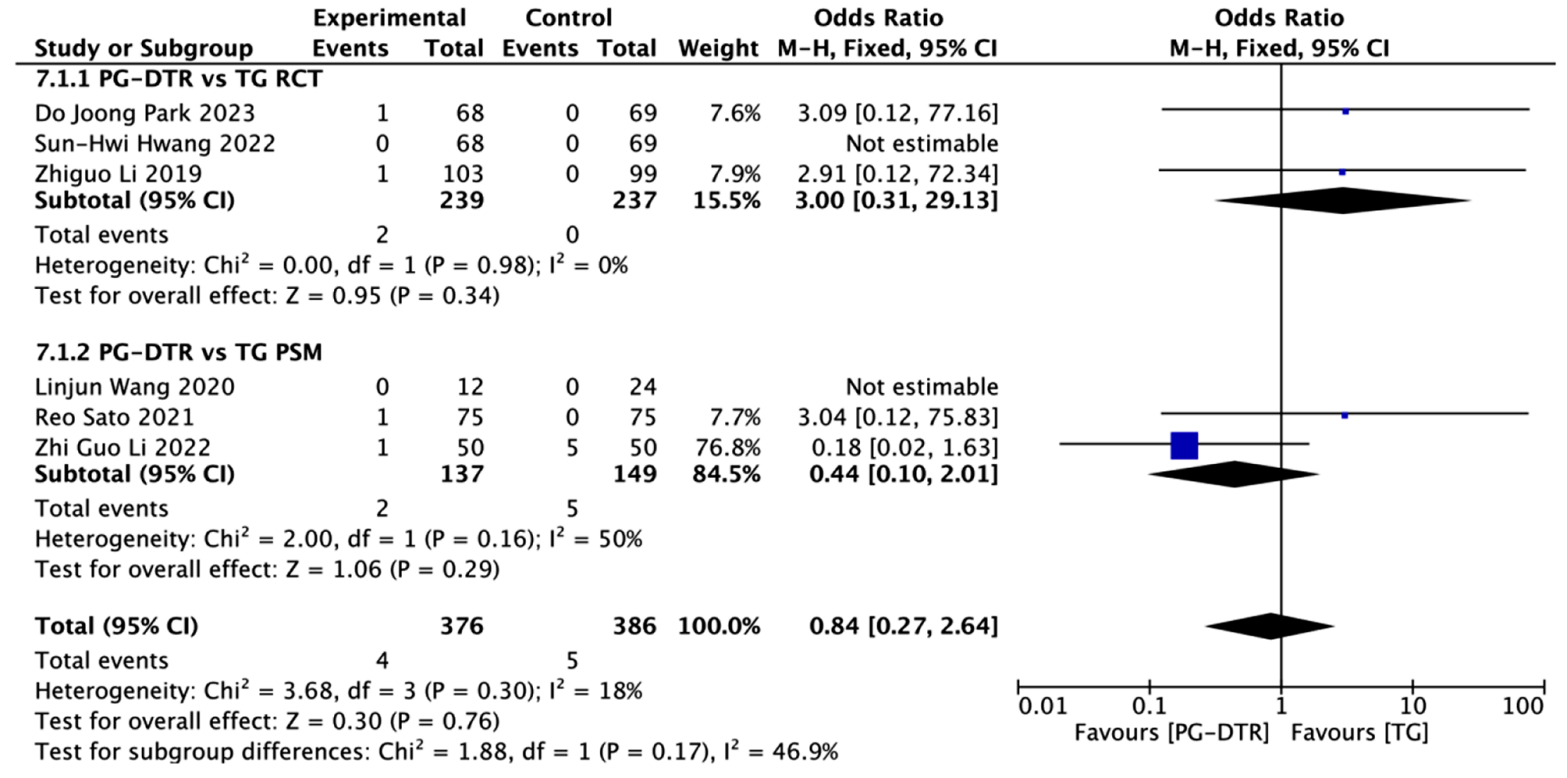
Forest plots of comparision between PG-DTG and TG-RY on postoperative mortality

A total of 762 patients in 6 papers were meta-analyzed in terms of Postoperative mortality [6–9, 13, 14, 16]. The results from the combined literature showed that PG-DTR did not have a significant advantage over TG in Postoperative mortality (OR = 0.84, 95% CI: 0.27 ~ 2.64, *P* = 0.76). Heterogeneity test (*P* = 0.03, I^2^ = 18%). (Fig. 8).

#### Results of the meta-analysis of the primary outcomes

**Fig. 9:**
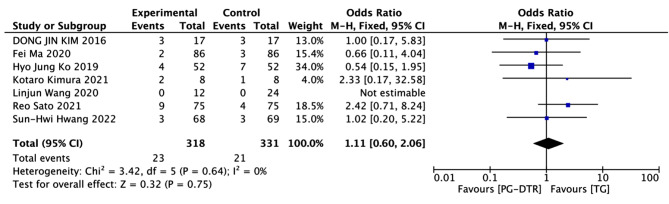
Forest plots of comparision between PG-DTG and TG-RY on ≥ Grade III C-D score

A total of 649 patients in 11 papers were meta-analyzed in terms of ≥ Grade III C-D score [7, 9–14]. The results from the combined literature showed that PG-DTR did not have a significant advantage over TG in ≥ Grade III C-D score (OR = 1.11, 95% CI: 0.60 ~ 2.06, *P* = 0.75). Heterogeneity test (*P* = 0.64, I^2^ = 0%). (Fig. 9).

**Fig. 10:**
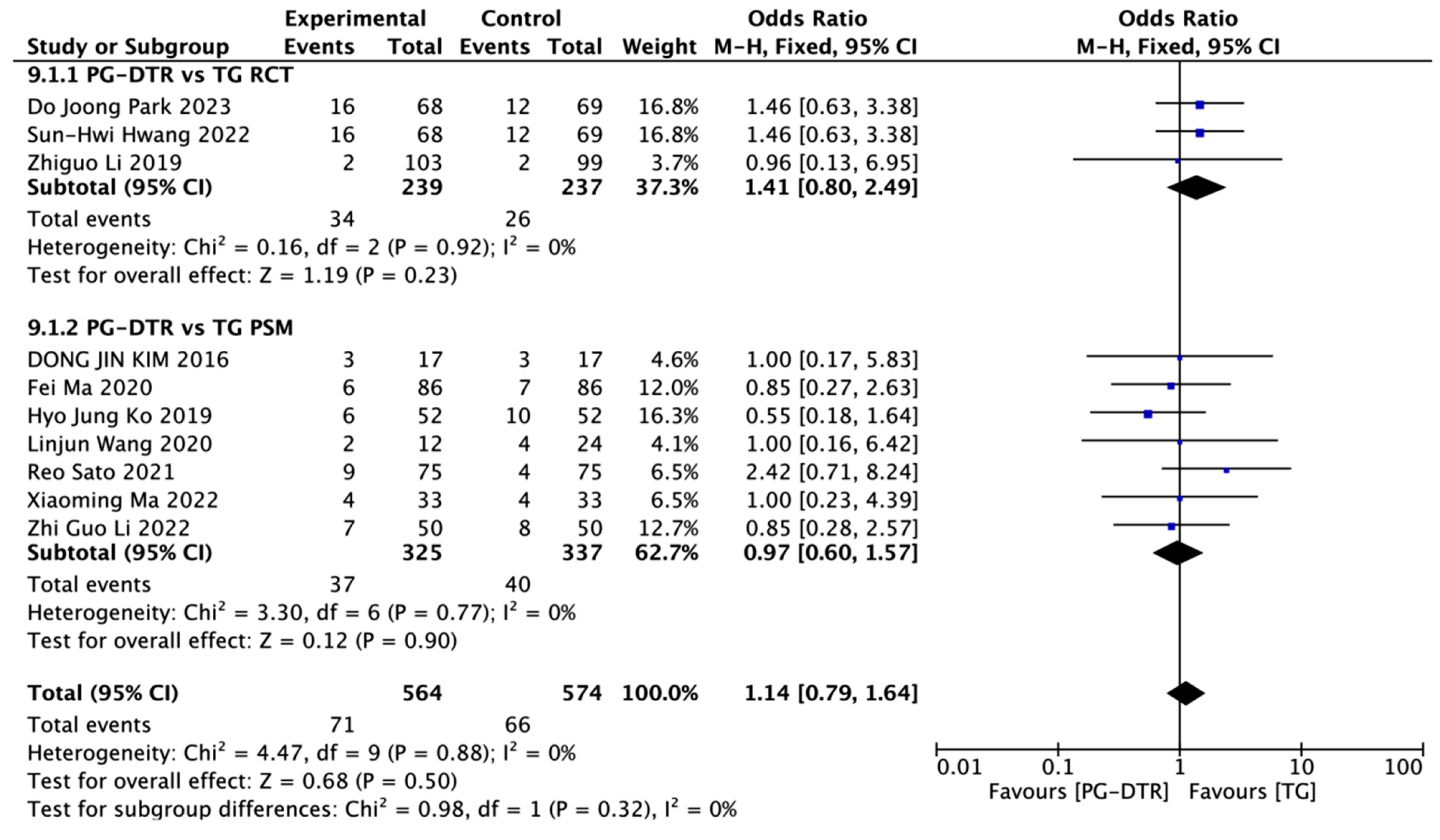
Forest plots of comparision between PG-DTG and TG-RY on early-phase complications

A total of 1138 patients in 10 papers were meta-analyzed in terms of early-phase complications.

 [6–11, 13–16]. The results from the combined literature showed that PG-DTR did not have a significant advantage over TG in early-phase complications (OR = 1.14, 95% CI: 0.79 ~ 1.64, *P* = 0.50). Heterogeneity test (*P* = 0.88, I^2^ = 0%). (Fig. 10).

**Fig. 11:**
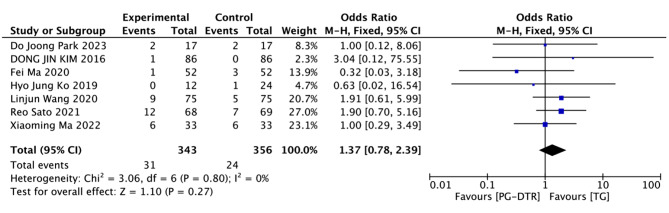
Forest plots of comparision between PG-DTG and TG-RY on late-phase complications

A total of 699 patients in 7 papers were meta-analyzed in terms of Late-phase complications [6, 9–11, 13–15]. The results from the combined literature showed that PG-DTR did not have a significant advantage over TG in Late-phase complications(OR = 1.37, 95% CI: 0.78 ~ 2.39, *P* = 0.27). Heterogeneity test (*P* = 0.80, I^2^ = 0%). (Fig. 11).

**Fig. 12:**
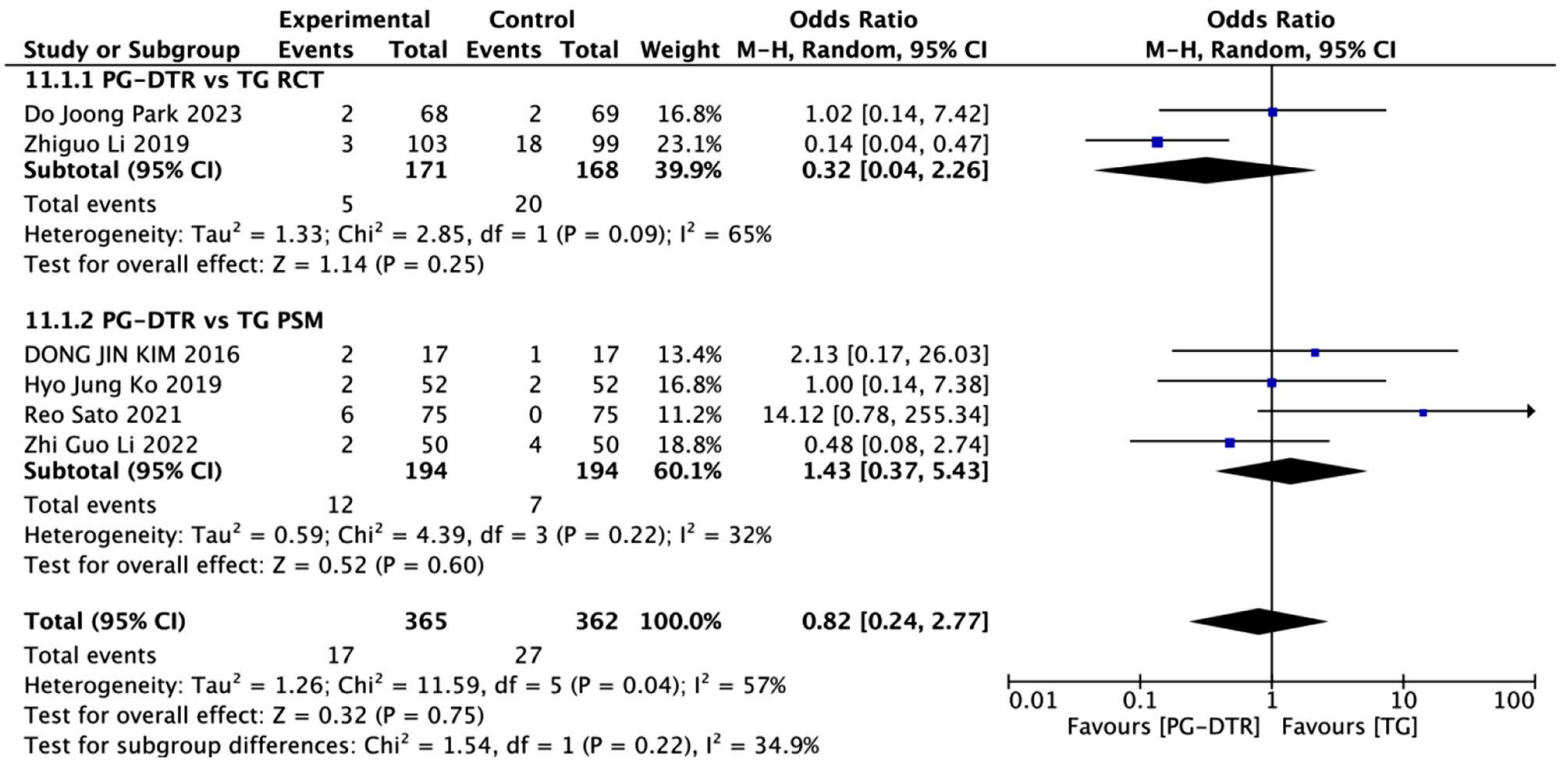
Forest plots of comparision between PG-DTG and TG-RY on the incidence of endoscopic gastroesophageal reflux

A total of 727 patients in 6 papers were meta-analyzed in terms of the incidence of endoscopic gastroesophageal reflux [6, 8, 9, 11, 14, 16]. The results from the combined literature showed that PG-DTR did not have a significant advantage over TG in the incidence of endoscopic gastroesophageal reflux(OR = 0.82, 95% CI: 0.24 ~ 2.77, *P* = 0.75). Heterogeneity test (*P* = 0.04, I^2^ = 57%). (Fig. 12).

**Fig. 13:**
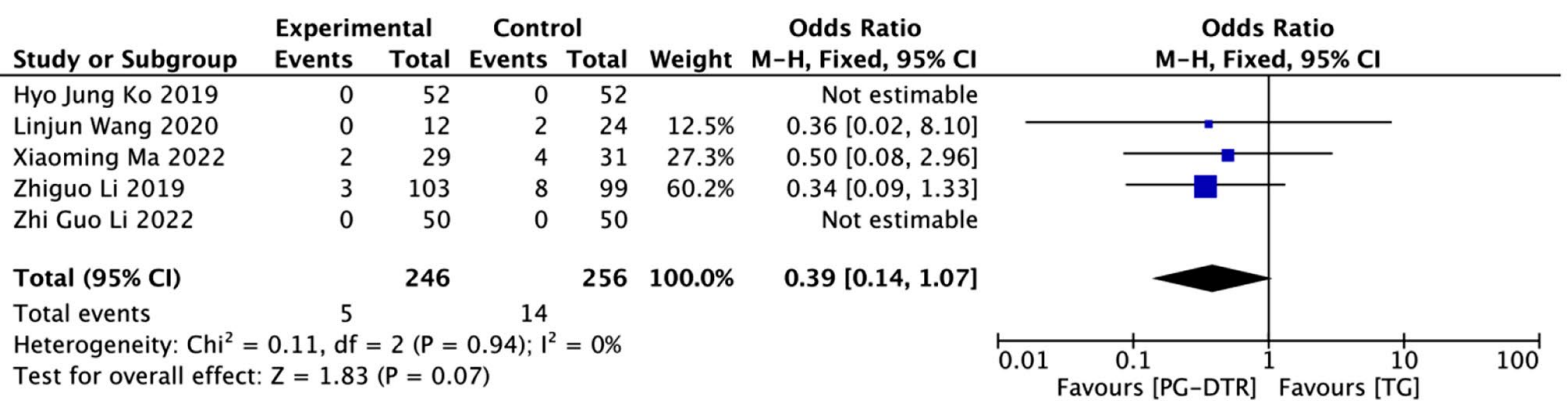
Forest plots of comparision between PG-DTG and TG-RY on Gastroesophageal reflux symptom evaluation (Visick score ≥ III)

A total of 502 patients in 5 papers were meta-analyzed in terms of the Gastroesophageal reflux symptom evaluation (Visick score)(≥ III) [8, 11, 13, 15, 16]. The results from the combined literature showed that PG-DTR did not have a significant advantage over TG in the Gastroesophageal reflux symptom evaluation (Visick score)(≥ III)(OR = 0.39, 95% CI: 0.14 ~ 1.07, *P* = 0.07). Heterogeneity test (*P* = 0.94, I^2^ = 0%). (Fig. 13).

**Fig. 14:**
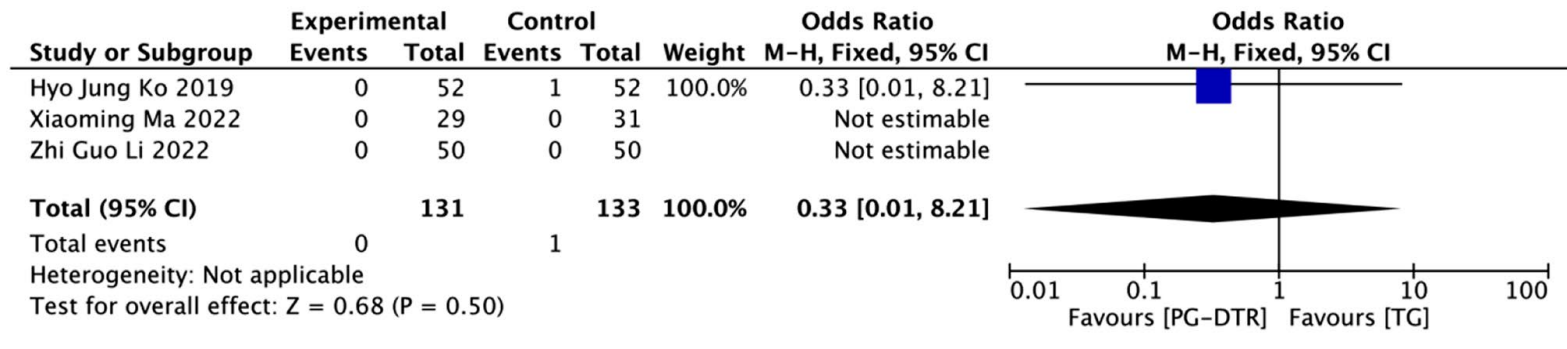
Forest plots of comparision between PG-DTG and TG-RY on Los Angeles classification(C or D)

A total of 264 patients in 3 papers were meta-analyzed in terms of the Los Angeles classification(C or D) [11, 15, 16]. The results from the combined literature showed that PG-DTR did not have a significant advantage over TG in the Los Angeles classification (C or D) (OR = 0.33, 95% CI: 0.01 ~ 8.21, *P* = 0.50). (Fig. 14).

**Fig. 15:**
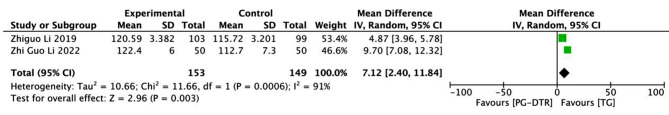
Forest plots of comparision between PG-DTG and TG-RY on Hemoglobin

A total of 302 patients in 2 papers were meta-analyzed in terms of hemoglobin [8, 16]. The results from the combined literature showed that PG-DTR had a significant advantage over TG in hemoglobin. (MD = 7.12, 95% CI: 2.40∼11.84 *P* = 0.003). Heterogeneity test (*P* = 0.0006, I^2^ = 91%). (Fig. 15).

**Fig. 16:**
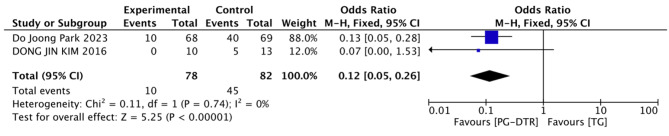
Forest plots of comparision between PG-DTG and TG-RY on receipt of vitamin B12 supplementation, No

A total of 160 patients in 2 papers were meta-analyzed in terms of the receipt of vitamin B12 supplementation [6, 9]. The results from the combined literature showed that PG-DTR did not have a significant advantage over TG in the receipt of vitamin B12 supplementation(OR = 0.12, 95% CI: 0.05 ~ 0.26, *P* < 0.00001). Heterogeneity test (*P* = 0.74, I^2^ = 0%). (Fig. 16).

**Fig. 17:**
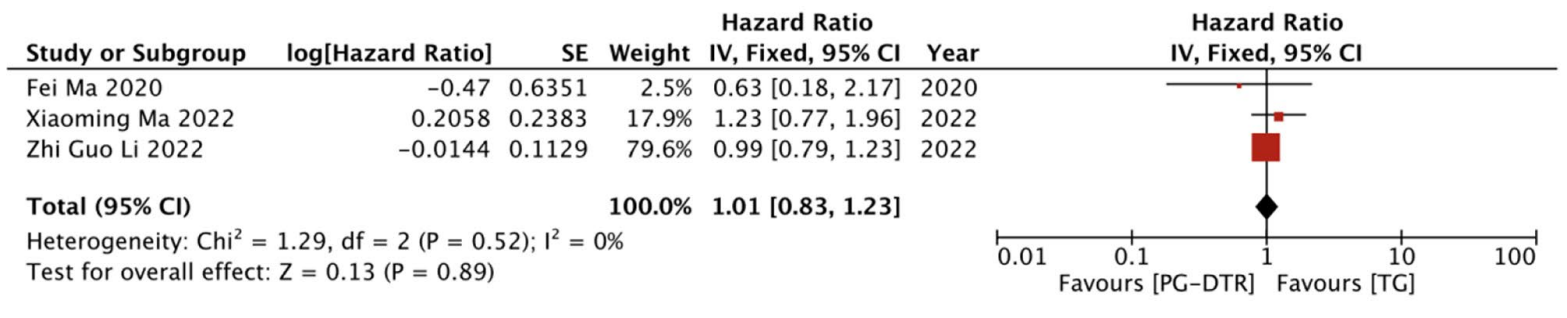
Forest plots of comparision between PG-DTG and TG-RY on the 5-year overall survival rates

A total of 338 patients in 3 papers were meta-analyzed in terms of the 5-year overall survival rates.

 [10, 15, 16].There is no significant difference between the proximal gastrectomy with double-tract reconstruction and the total gastrectomy reconstruction group in the 5-year overall survival rates(HR = 1.01, 95% CI: 0.83 ~ 1.23, *P* = 0.89). Heterogeneity test (*P* = 0.52, I^2^ = 0%). (Fig. 17).

### Sensitivity analysis and publication bias analysis

The findings of this study indicate a high degree of heterogeneity in the mean operation time, estimated blood loss, postoperative hospital stay, retrieved number of lymph nodes, time to gas-passing, the incidence of endoscopic gastroesophageal reflux, and Hemoglobin. Consequently, individual studies were systematically excluded and sensitivity analyses were conducted. The results demonstrate that the combination of postoperative hospital stay, retrieved number of lymph nodes, time to gas-passing, and Hemoglobin in this study is generally reliable. However, the stability of the results for mean operation time, estimated blood loss, and the incidence of endoscopic gastroesophageal reflux was found to be lacking. This may be attributed to potential selection bias (e.g., differences in patient inclusion/exclusion criteria and tumor staging), variations in surgical methods and skill levels among surgeons, and discrepancies in medical device specifications. To assess potential bias in this study with early complications incidence and 5-year overall survival rates as primary outcome measures, a funnel plot was constructed.The symmetric distribution of data points on the funnel plot suggests a low likelihood of publication bias in this meta-analysis (Fig. 17).

**Fig. 18:**
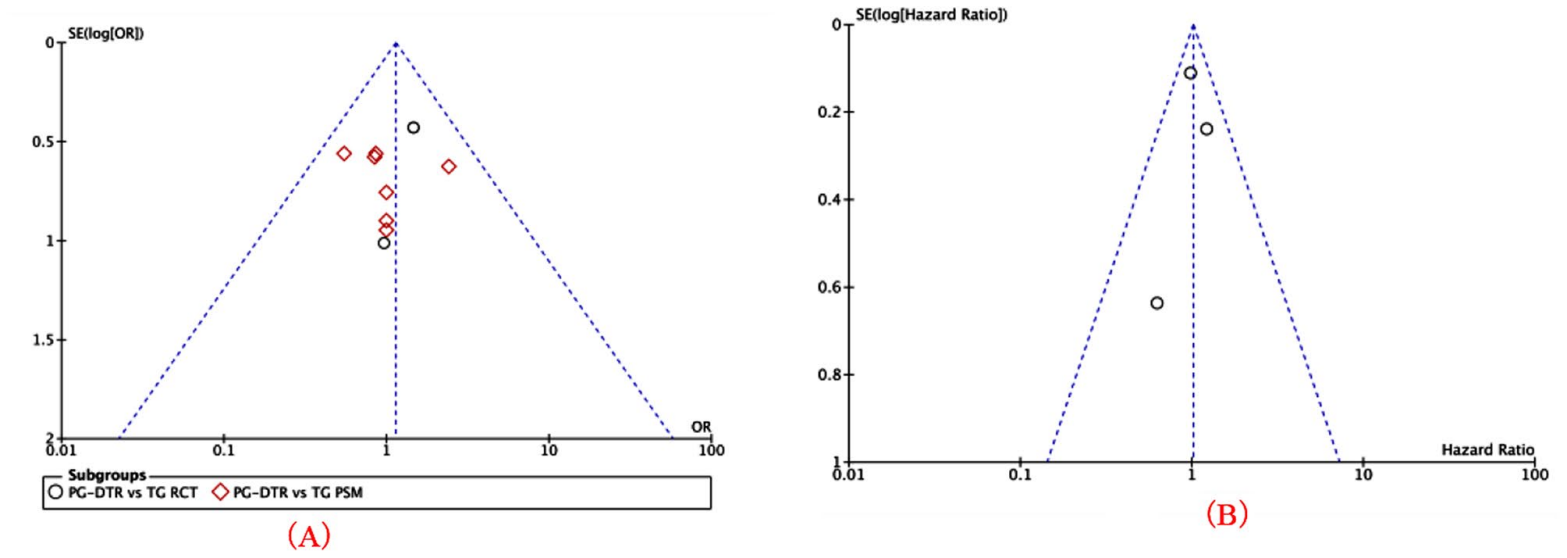
Funnel plots of each outcome. (A) Early-phase complications; (B)the 5-year overall survival rates

## Discussion

In recent years, the method of reconstructing the digestive tract after radical proximal gastrectomy with double-tract anastomosis has been increasingly utilized due to continuous exploration and attempts by numerous surgeons. However, a major issue associated with proximal gastrectomy is the high incidence of reflux esophagitis [17], which severely impairs the patient’s quality of life after surgery despite preservation of some stomach function. To address this problem, various reconstructive approaches have been attempted by surgeons in order to reduce the incidence of reflux esophagitis after proximal gastrectomy to a level comparable to total gastrectomy. Currently, there is no consensus on the selection of digestive tract reconstruction after proximal gastrectomy. Nevertheless, it is recommended that proximal gastrectomy double-tract anastomosis be considered due to the shift in surgical treatment concept from “extensive and standardized surgical resection” to “individualized and precise surgical resection.” The application of proximal gastrectomy double-tract anastomosis in treating proximal gastric cancer is mainly limited by concerns regarding surgical safety, tumor safety, postoperative quality of life, and nutritional status.

In terms of surgical safety, the results of this meta-analysis revealed no statistically significant differences between the two groups in estimated blood loss, postoperative hospital stay, gas flow, and postoperative mortality. However, it was found that PG-DTR operated longer than LTG, and this difference was deemed to be statistically significant. Kimura et al. [10] reported that proximal gastrectomy with double-tract required a shorter operating time than total gastrectomy with Roux-en-Y reconstruction. Although adding gastrojejunal anastomosis to PG-DTR theoretically leads to a longer surgical time, it is important to note that total gastrectomy and D2 lymph node dissection also increase the surgical time. Therefore, further high-quality RCT studies are needed for additional validation in the future. The results of this meta-analysis indicated no significant differences in the incidence of serious complications (≥ Grade III C-D score), Early-phase complications, and Late-phase complications between the two groups. Gastroesophageal reflux is a common complication following proximal gastrectomy, and several studies have demonstrated that PG-DTR (proximal gastrectomy with double-tract reconstruction) has a definite anti-reflux effect. Chio et al [18] conducted an analysis on the efficacy of 37 patients who underwent total laparoscopic proximal gastrectomy with double-channel reconstruction. All patients underwent successful postoperative endoscopic monitoring, and none of them developed reflux esophagitis or stenosis after surgery. The findings of this meta-analysis also suggest that PG-DTR has a superior anti-reflux effect and a lower incidence of postoperative complications.

Ensuring the safety of tumor resection and determining the optimal resection range primarily involves two key aspects: “margin distance” and “lymph node dissection [19]. Adequate resection margins are crucial in establishing the resection line for curative gastrectomy. We have identified current issues related to margin distance in stage, infiltrative, signet ring cell, and undifferentiated carcinoma cases [19]. The following discussion focuses on margin distance for Stage and these pathological types: (1) Staging of Gastric Cancer and Margin Distance: In early stage gastric cancer (T1), the tumor is confined to the mucosal or submucosal layer, allowing for a relatively small margin distance. For T1 tumors, a gross resection margin of 2 cm should be achieved. However, for T2 stage and higher gastric cancer where the tumor may have penetrated deeper layers, it is necessary to increase the margin distance accordingly to ensure complete removal of the tumor [19]. (2) Infiltration and Margin Distance: In cases of invasive gastric cancer, tumor cells may extensively infiltrate surrounding tissue, posing challenges for surgical intervention and creating uncertainty regarding the scope of resection. In such instances, it is advisable to determine an appropriate distance from the incisal margin based on preoperative imaging examination results, endoscopic biopsy findings, and intraoperative exploration. Generally, for invasive gastric cancer, a recommended incisal margin distance of at least 3-5 cm from the tumor margin is advised to ensure complete tumor resection [20].(3) Signet ring cell carcinoma and Margin distance. Signet ring cell carcinoma is a highly malignant type of gastric cancer with unique pathological features. Due to the abundant mucus within the tumor cells, the boundaries between the cells are indistinct, making it challenging to ascertain the cleanliness of the incisal margin during surgery. Therefore, more stringent criteria for margin distance are recommended for resection of signet ring cell carcinoma. Specifically, it is advised that the incisal margin should be at least 5 cm from the tumor margin, and frozen sections should be performed during the operation to ensure a negative incisal margin [21]. (4) Undifferentiated cancer and Margin distance. Undifferentiated cancer is characterized by the highest degree of malignancy, low differentiation of tumor cells, rapid growth, and easy metastasis. Therefore, caution should be exercised in approaching resection scope for undifferentiated cancer and it should be more extensive. It is recommended that the distance from the tumor margin to the incisal margin should be at least 5-7 cm, and thorough lymph node dissection should be performed during the operation [22].The safety of tumor resection in the context of gastric lymph node dissection is a topic of controversy. Compared to total gastrectomy, lymph node dissection with proximal gastrectomy has a more limited scope, as it does not include stations no.4d, 5, 6, and 12a. A meta-analysis showed that fewer lymph nodes were removed during surgery in the proximal gastrectomy double-tract group compared to the total Roux-en-Y gastrectomy group [10]. Yura et al. reported very low lymph node metastasis rates for stage cT2-3 proximal gastric cancer: 0.99% for group 4d, 0% for group 5, 0% for group 6, and 0.006% for group 12a [23]. This suggests that lymph node dissection may not be necessary for stage T2-3 proximal gastric cancer. Recent clinical studies from Japan also support the feasibility of proximal gastrectomy for locally advanced gastric cancer with cT2-T4. In terms of postoperative survival rate, multi-center studies by domestic and foreign scholars have shown no statistical difference in the 5-year survival rate after proximal gastrectomy and total gastrectomy for upper gastric cancer [10]. The meta-analysis also found no statistical difference in the 5-year postoperative survival rate between the two groups, supporting the conclusion that proximal gastrectomy with double-tract reconstruction can ensure tumor safety for patients with early or even advanced proximal gastric cancer after surgery.

Some indicators reflecting nutritional status were also included in this meta-analysis. The hemoglobin level at 1 year after proximal gastrectomy and double-tract anastomosis was significantly higher than in the Roux-en-Y group, indicating a clear advantage of this surgical approach. Proximal gastrectomy with double-tract reconstruction allows for food to enter the distal jejunum from both the residual stomach and jejunum, respectively. This mechanism promotes increased meal volume for patients, as well as improved food storage and mixing within the residual stomach, leading to better nutrient absorption. Additionally, the passage of food through the jejunum helps maintain gastrointestinal hormone balance and enhances iron and vitamin B12 absorption. Furthermore, endogenous factor is a glycoprotein secreted by parietal cells of gastric mucosa that plays a crucial role in vitamin B12 absorption. Proximal gastrectomy with double channels retains some ability to secrete internal factors, which supports vitamin B12 absorption and erythropoiesis. Compared to total gastrectomy, proximal gastrectomy with double-tract can help prevent pernicious anemia to a certain extent.

There are still some limitations in this meta-analysis. Firstly, out of the 11 literature included, only 3 were RCTs while the rest were PSMs. Secondly, PSMs themselves have certain design flaws. Thirdly, most of the qualified literature was submitted by Asian scholars, indicating a regional bias. Lastly, only English-language literature was included, which may introduce linguistic bias.

## Conclusions

Proximal gastrectomy with double-tract reconstruction is considered a safe and feasible treatment option for upper gastric carcinoma. Although the operation time was slightly longer in the proximal gastrectomy with double-tract reconstruction group compared to the total gastrectomy group, both groups showed comparable rates of serious complications, short-term complications, long-term complications, and quality of life. Furthermore, there was no significant difference in the 5-year overall survival rates between the two groups, indicating reliable long-term tumor safety. Additionally, the proximal gastrectomy with double-tract reconstruction group exhibited higher long-term hemoglobin levels, lower likelihood of requiring vitamin B12 supplementation, and better long-term efficacy when compared to the total gastrectomy group. In conclusion, proximal gastrectomy with double-tract reconstruction is considered one of the more rational surgical approaches for upper gastric cancer.

## Data Availability

All data generated or analysed during this study are included in this published article.
